# Emergence of Pediatric Melioidosis in Siem Reap, Cambodia

**DOI:** 10.4269/ajtmh.2010.10-0030

**Published:** 2010-06

**Authors:** Yos Pagnarith, Varun Kumar, Janjira Thaipadungpanit, Vanaporn Wuthiekanun, Premjit Amornchai, Lina Sin, Nicholas P. Day, Sharon J. Peacock

**Affiliations:** Angkor Hospital for Children, Siem Reap, Cambodia; Mahidol-Oxford Tropical Medicine Research Unit, Faculty of Tropical Medicine, Mahidol University, Bangkok, Thailand; Department of Microbiology and Immunology, Faculty of Tropical Medicine, Mahidol University, Bangkok, Thailand; Center for Clinical Vaccinology and Tropical Medicine, Nuffield Department of Clinical Medicine, University of Oxford, Churchill Hospital, Oxford, United Kingdom; Department of Medicine, University of Cambridge, Addenbrooke's Hospital, Cambridge, United Kingdom

## Abstract

We describe the first cases of pediatric melioidosis in Cambodia. Thirty-nine cases were diagnosed at the Angkor Hospital for Children, Siem Reap, between October 2005 and December 2008 after the introduction of microbiology capabilities. Median age was 7.8 years (range = 1.6–16.2 years), 15 cases were male (38%), and 4 cases had pre-existing conditions that may have pre-disposed the patient to melioidosis. Infection was localized in 27 cases (69%) and disseminated in 12 cases (31%). Eleven cases (28%) were treated as outpatients, and 28 (72%) cases were admitted. Eight children (21%) died a median of 2 days after admission; seven deaths were attributable to melioidosis, all of which occurred in children receiving suboptimal antimicrobial therapy and before bacteriological culture results were available. Our findings indicate the need for heightened awareness of melioidosis in Cambodia, and they have led us to review microbiology procedures and antimicrobial prescribing of suspected and confirmed cases.

## Introduction

Melioidosis is a serious infectious disease caused by the Gram-negative bacterium *Burkholderia pseudomallei*.^1^,^2^ This organism is present in the environment in a defined geographic distribution including much of south and east Asia, northern Australia, and areas of South America, where infection is thought to be acquired after bacterial inoculation, ingestion, or inhalation.^1^–^3^ The clinical presentation is highly variable and ranges from a mild localized infection to acute fulminant sepsis with widespread bacterial dissemination.^1^,^2^ The clinical diagnosis of melioidosis is notoriously inaccurate, and diagnostic confirmation relies on culture of *B. pseudomallei*.^4^ Lack of microbiological services in many of the countries predicted to be affected by melioidosis is likely to result in gross underreporting of cases and an underestimate of the global burden of this infection.

Melioidosis is a leading cause of bacterial sepsis in northeast Thailand.^5^–^8^ Childhood infection accounts for around 10% of cases overall in this setting, and acute suppurative parotitis accounts for one-third of pediatric cases.^9^ Adjacent to northeast Thailand is Laos to the east and southeast and Cambodia to the south. Melioidosis was first reported from Laos in 2001 after the development of a diagnostic microbiology laboratory at Mahosot Hospital, Vientiane,^10^ and *B. pseudomallei* was subsequently isolated from the surrounding environment.^11^ *B. pseudomallei* has also been isolated from rice paddies in Siem Reap province in northwest Cambodia,^12^ and a recent report of two cases of melioidosis in adults from southern Cambodia has confirmed the presence of melioidosis in the indigenous population.^13^ A seroprevalence study of children presenting to a hospital in Siem Reap detected antibodies to *B. pseudomallei* in 16% of cases,^12^ but melioidosis has not been detected previously in this pediatric population. Here, we report the identification of 39 cases of melioidosis at the Angkor Hospital for Children in Siem Reap, the first reported cases in Cambodian children. Their identification followed the introduction of diagnostic microbiology capabilities, highlighting one of the many benefits of laboratory strengthening in this region.

## Materials and Methods

### Setting and patients.

The study was conducted at the Angkor Hospital for Children (AHC), an non-governmental organization (NGO)-funded teaching hospital in Siem Reap, situated in the province of Siem Reap, northwest Cambodia. The AHC provides free outpatient, inpatient, emergency, surgical, medical, ophthalmological, and dental care, and it maintains 50 inpatient beds spread across high-, medium-, and low-intensity care areas. The outpatient department sees an average of approximately 400 children each day from an unrestricted catchment area.

The study protocol was reviewed and approved by the Ethical Review Board of the Angkor Hospital for Children. A laboratory-based study was conducted between October 2005 and December 2008 to identify children presenting to inpatient or outpatient departments with one or more microbiological samples positive for *B. pseudomallei*. A retrospective review of case records was performed at the end of this period to collect information on age, gender, area of residence, sample(s) positive for *B. pseudomallei*, known sites of organ involvement, results of laboratory and radiological investigations, antimicrobial treatment, procedures, duration of hospital stay, outcome at hospital discharge, and details of follow-up. Sites of infection were established based on a record of history and examination findings in the medical notes together with investigation reports and procedure notes.

Melioidosis was classified as localized or disseminated. Localized infection was defined as a single, discrete, culture-positive focus of infection in the absence of a positive blood culture or clinical and/or microbiological evidence of dissemination to a second site. Disseminated infection was defined as the presence of infection in two or more discrete body sites and/or the presence of *B. pseudomallei* in blood.

### Microbiological methods.

Specimens from patients with suspected melioidosis were processed using standard laboratory procedures with the addition of Ashdown selective agar for culture of samples from colonized sites.^14^ Identification of *B. pseudomallei* was made on the basis of Gram stain, resistance to gentamicin and colistin, a positive latex agglutination test,^15^,^16^ and the API 20NE profile.^16^ Susceptibility testing was performed by disk diffusion assay for ceftazidime, meropenem, amoxicillin-clavulanate, and doxycycline and by E-test for trimethoprim-sulfamethoxazole (TMP-SMX). Isolates were stored in Tryptone Soya broth (TSB) with 20% glycerol at −80°C.

### Molecular characterization of *B. pseudomallei*.

Genomic DNA was extracted from a 1-mL overnight culture of *B. pseudomallei* with an optical density of 1.0 at 600 nm using the High Pure PCR Template Preparation Kit (Roche Applied Science, Mannheim, Germany). Multilocus sequence typing (MLST) was performed as described previously.^17^ Sequence-type (ST) assignment was based on the sequence of the alleles at each locus of seven housekeeping genes using the MLST database (www.mlst.net). Sequences that were not in the database were checked by resequencing, and then assigned as new alleles and deposited in the MLST allele database.

MLST was performed on 39 *B. pseudomallei* isolates cultured from children with melioidosis described in this study and 14 *B. pseudomallei* isolates cultured previously from soil in Cambodia, of which 12 were isolated in Siem Reap province during 2006^12^ and 2 were isolated in Phnom Penh in southern Cambodia in 1996. MLST data on *B. pseudomallei* isolates from Thailand that had originated in our laboratory and/or had been reported previously^17^–^19^ were downloaded from www.mlst.net (*N* = 462). Neighbor-joining trees were reconstructed using the two-parameter method of distance estimation as implemented in MEGA 4.0.

## Results

### Overview of cases.

**Figure 1.**
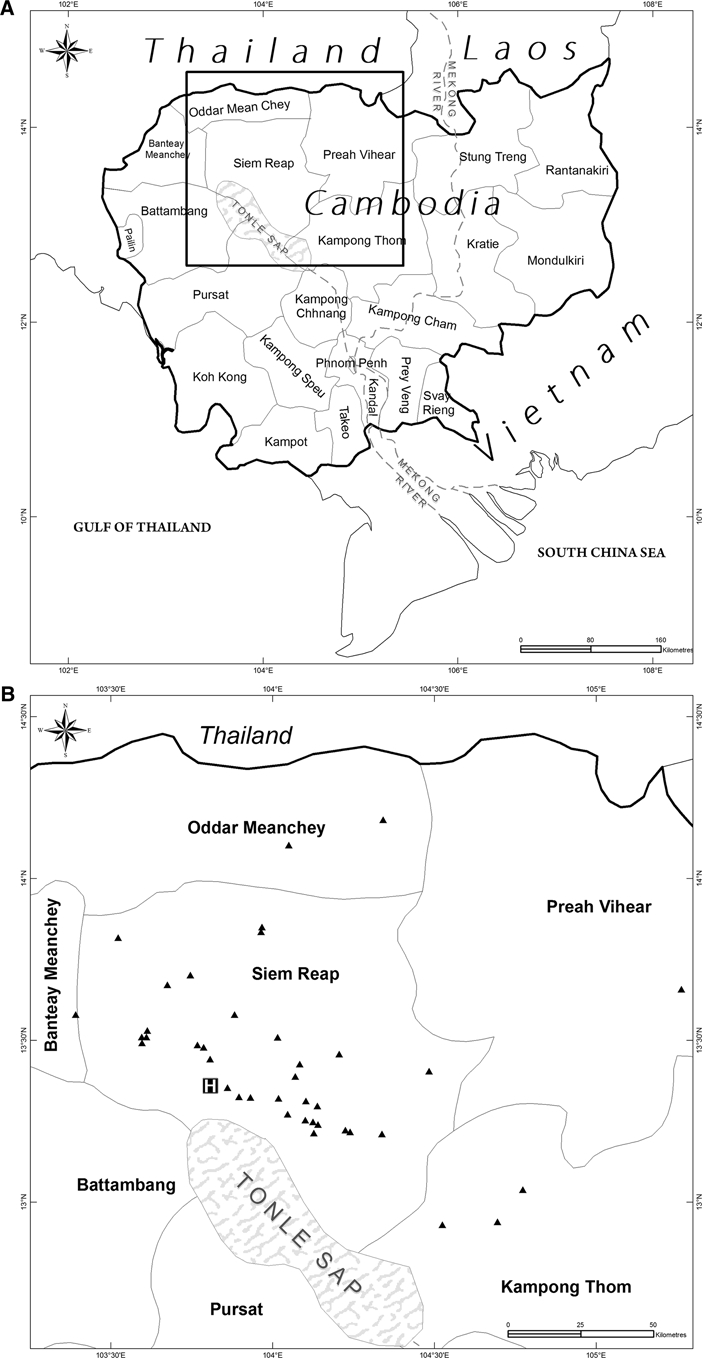
Whole country map (**A**) and zoomed view showing the place of residence (triangles) of 39 children with melioidosis presenting to the Angkor Hospital for Children (**B**). H denotes this hospital.

Thirty-nine cases of culture-proven melioidosis were identified in children presenting to the AHC between October 2005 and December 2008. No case clustering was observed, and cases were distributed throughout the study period as follows: 2005 (3 months), *N* = 2; 2006, *N* = 9; 2007, *N* = 13; 2008, *N* = 14. The geographic location of the village or town in which the cases lived is shown in Figure 1. These observations suggest that infections were sporadic and not related to one or more outbreaks.

Summary data for the 39 cases are shown in Table 1. Median age was 7.8 years (range = 1.6–16.2 years), and the male/female ratio was 1:1.6. Four children (10%) had pre-existing conditions that may have pre-disposed them to infection: juvenile rheumatoid arthritis (1 case), thalassemia and renal disease (1 case), Pott's disease (tuberculosis of the spine; 1 case), and chronic otitis media with *B. pseudomallei* meningitis (1 case).

Infection was defined as localized in 27 children (69%) and disseminated in 12 children (31%); 21 of 27 localized infections (78%) were in the region of the head and neck, including 15 cases of acute suppurative parotitis. The disseminated infection group included nine children with blood cultures positive for *B. pseudomallei* in association with clinical and radiographic evidence of pneumonia (*N* = 2), clinical meningitis and a history of chronic suppurative otitis media for whom the cerebrospinal fluid (CSF) culture was negative (*N* = 1), or no other identified focus of infection (*N* = 6) and three children who did not have a positive blood culture with multiple skin pustules (*N* = 1), mastoiditis and abscesses in the liver and spleen on ultrasound (*N* = 1), or pneumonia associated with respiratory failure and septic shock (*N* = 1). This last case with respiratory secretions positive for *B. pseudomallei* had neither positive blood cultures nor a second site of infection, but was classified as having disseminated infection based on the systemic response and severity of illness.

Eight of thirty-nine children (21%) died a median of 2 days after admission (range, day of admission to day 23), all of whom were in the disseminated infection group (8/12; 67%). Seven deaths were attributable to melioidosis, whereas the eighth case had both tuberculosis meningitis and *B. pseudomallei* pneumonia and had responded to 14 days of ceftazidime by the time of death on day 23 because of intracranial hypertension secondary to tuberculosis meningitis. A positive blood culture was associated with a very poor prognosis, and death occurred in 7 of 9 cases (78%). Both children with a total white blood cell (WBC) count of less than 5.0 × 10^9^/L (1.6 × 10^9^/L and 2.6 × 10^9^/L, respectively) died.

All 39 *B. pseudomallei* isolates were susceptible to ceftazidime, meropenem, amoxicillin-clavulanate, TMP-SMX, and doxycycline, the most common drugs used for the treatment of adult melioidosis.

### Clinical management.

Eleven children (28%) were treated in the outpatient department alone for localized *B. pseudomallei* infection (parotid abscess, 4; superficial soft-tissue abscess, 7). Incision and drainage of pus was performed in 10 of these cases, and all 11 cases were prescribed one or more antimicrobial drugs for a median of 4 weeks (range, 5 days to 17 weeks; IQR = 1–6 weeks). The antimicrobial drugs prescribed were amoxicillin-clavulanate alone (*N* = 2), amoxicillin-clavulanate in combination with TMP-SMX (*N* = 2), amoxicillin-clavulanate in combination with ciprofloxacin (*N* = 2), or cloxacillin alone (*N* = 5). Six of eleven children did not attend for follow-up, and their outcome was unknown. Five children followed-up for a period of 4–17 weeks were documented as having resolution of clinical features of infection.

Twenty-eight children (72%) were admitted to the AHC; 16 cases had localized infection, and 12 cases had disseminated infection. Duration of admission for the 20 in-patient survivors ranged from 1 to 31 days (median = 15.5 days; IQR = 12.0–17.5 days). Management is presented separately for localized and disseminated infection.

### Admitted with localized infection (*N* = 16).

All cases had an incision and drainage procedure. Fifteen cases were started on empiric parenteral antimicrobial drugs (ceftriazone or cloxacillin) for suspected localized bacterial infection at the time of admission. The remaining case was a child with a pharyngeal abscess who was treated with incision and drainage under general anesthetic and discharged the following day with a 7-day course of oral cloxacillin for suspected *S. aureus* infection before culture results were available. This child was lost to follow-up. The remaining 15 cases were still in the hospital when the *B. pseudomallei* culture result became available, and therapy was changed to ceftazidime (*N* = 14) or parenteral amoxicillin-clavulanate (*N* = 1) for a median of 11 days (range = 1–15 days; IQR = 9–14 days). A switch to oral antibiotics was made prior to (*N* = 5) or on the day of discharge (*N* = 10). All 15 children were discharged on oral amoxicillin-clavulanate, which was prescribed for a median of 7 weeks (range = 2–23 weeks; IQR = 4–13 weeks). One or more follow-up appointments were attended by 13 of 15 cases during antimicrobial therapy, and clinical features of infection were documented to have resolved in all of these cases.

### Admitted with disseminated infection (*N* = 12).

The seven attributable deaths in this group occurred before the culture results were available, and these patients were receiving ceftriaxone at the time of death. All 7 cases were admitted to the intensive care unit where management included ventilation for respiratory failure (*N* = 1), inotropes for circulatory failure (*N* = 2), or both treatment modalities (*N* = 4). Of note, two of four survivors in the disseminated group also had ventilatory failure requiring ventilation (1 case) or continous positive airway pressure (CPAP) therapy (1 case), and one of these cases also required inotropes. Empiric antimicrobial therapy (ceftiaxone) was also given to the four survivors before culture results, and after culture results were known, this therapy was followed by parenteral ceftazidime (1 case), amoxicillin-clavulanate plus ceftriaxone (2 cases), or ciprofloxacin (1 case). The duration of parenteral therapy in this group ranged from 10 to 31 days. Oral amoxicillin-clavulanate was given to all four cases after discharge and continued for between 5 and 23 weeks. One or more follow-up appointments were attended by the four cases during this treatment period, and clinical features of infection were documented to have resolved in all of these cases.

### Bacterial genotyping.

The 39 *B. pseudomallei* isolates from the study patients were resolved by MLST into 33 different STs (0.85 ST per isolate). Of these, 28 STs occurred one time, 4 STs occurred two times, and one ST (ST 70) occurred three times. ST 70 is the most frequent clone isolated in Thailand and has also been identified previously in Laos and Hong Kong. Fourteen STs (42%) had not been identified previously and were unique to this study, one of which also included a novel allele at *gmhD*. The remaining 19 STs have been described previously in relation to isolates obtained from the environment and/or human disease in at least one Asian country.

Fourteen *B. pseudomallei* isolates cultured previously from soil in Cambodia were also examined using MLST, of which 12 were isolated in Siem Reap province during 2006^12^ and 2 were isolated in Phnom Penh in southern Cambodia in 1996. These were resolved into 14 different STs, including an additional 6 unique STs that have not been described previously and were not represented in the invasive-strain collection described above. Of the remainder, four STs were also represented in the collection of 39 pediatric isolates, and four STs were not found in the pediatric collection but had been isolated previously in Asia.

**Figure 2.**
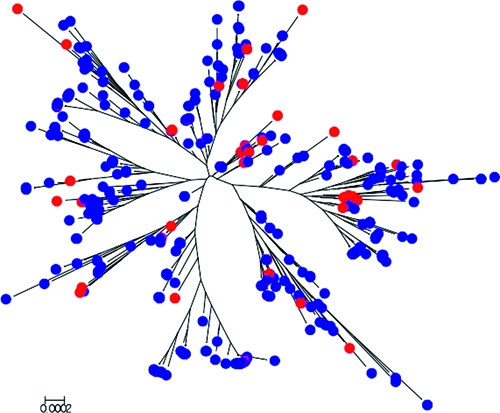
Neighbor-joining tree using concatenated sequences of all seven loci for Cambodian *B. pseudomallei* isolates from pediatric cases or soil (*N* = 53) together with concatenated sequences downloaded from the MLST website (www.mlst.net) for *B. pseudomallei* isolates from Thailand that had originated in our laboratory and/or had been reported previously^18^–^20^ (*N* = 462). Red circles denote Cambodian isolates, and blue circles denote Thai isolates.

The large number of STs found in this evaluation (including a total of 20 novel STs) is consistent with studies elsewhere in Asia and Australia,^17^–^20^ and is based on a high rate of recombination of a pool of largely known alleles. A neighbor-joining tree was constructed using concatenated sequences of all seven loci for Cambodian invasive and soil isolates (*N* = 53) together with concatenated sequences downloaded from the MLST website (www.mlst.net) for *B. pseudomallei* isolates from Thailand that had originated in our laboratory and/or had been reported previously^17^–^19^ (*N* = 462). This showed that isolates from Cambodia and Thailand were highly related (Figure 2).

## Discussion

This is the first description of pediatric melioidosis in Cambodia. It was predicted that melioidosis would occur in our patient population, because the incidence of melioidosis in neighboring northeast Thailand is high.^5^–^8^ *B. pseudomallei* has been isolated from soil in the province of Siem Reap,^12^ and seropositivity in the pediatric population has been already documented.^12^ However, we are able to draw several important lessons from our study.

This report highlights the value of diagnostic microbiology in defining the epidemiology of infectious diseases in developing world settings. Patterns of infectious diseases and resistance rates of prevalent pathogens may vary both within and between countries, and microbiology provides relevant, region-specific information on which to base effective empiric antimicrobial prescribing policies. Information generated by diagnostic microbiology also provides a feedback loop to physicians on the accuracy of presumptive admission diagnoses. Melioidosis emerges when diagnostic microbiology facilities become available in areas where *B. pseudomallei* is present in the environment, but this is just one of many examples of the benefits associated with culture capabilities at the AHC. For example, we have recently identified children infected with multi-resistant community-associated methicillin resistant *Staphylococcus aureus* (CA-MRSA)^21^ and are currently defining rates of CA-MRSA carriage in the community. Our laboratory is also providing information for the region on the burden of childhood disease from vaccine-preventable infections such as those caused by *Streptococcus pneumoniae* and *Haemophilus influenzae*.

The empiric antimicrobial drug of choice for children admitted to the AHC with suspected bacterial infection is ceftriaxone. This has broad-spectrum activity against a wide range of Gram-positive and Gram-negative bacteria, but is not recommended for melioidosis. Current guidelines for the antimicrobial therapy of melioidosis are based on trials conducted in adult patients.^22^–^24^ They consist of an intensive phase of intravenous antimicrobial agents (ceftazidime or a carbapenem drug) for a minimum of 10–14 days followed by an eradication phase of oral antimicrobial agents (TMP-SMX with or without doxycycline or amoxicillin-clavulanate for pregnant women, children, or adults who cannot tolerate TMP-SMX) to complete a course of treatment lasting 12–20 weeks. A systematic evaluation of the antimicrobial therapy of children has not been published. This is an important knowledge gap, because localized infection may be cured with shorter courses of antibiotics, particularly when infection is mild and localized and a procedure is performed to drain pus.

Ceftriaxone and other cephalosporins, including cefotaxime, are active against *B. pseudomallei in vitro*. In a study of 100 *B. pseudomallei* isolates from northern Australia, the minimum inhibitory concentration (MIC)_50_ and MIC_90_ were 4 µg/mL and 8 µg/mL, respectively, with a MIC range of 2–8 µg/mL^25^ (MIC for susceptible Gram-negative bacteria is ≤ 8 µg/mL). The comparative MIC values for ceftazidime in the same study were 2 µg/mL and 4 µg/mL for the MIC_50_ and MIC_90_, respectively, with a range of 1–8 µg/mL^25^ (MIC for susceptible *B. pseudomallei* is ≤ 8 µg/mL).^26^ This suggests that ceftazidime may have an advantage over ceftriaxone *in vivo*. The dose of ceftriaxone used at the AHC is 50–75 mg/kg given one time daily or in two divided doses. A possible disadvantage of one-time daily dosing is that the plasma concentration may fall below the MIC of the organism; average reported plasma concentrations in healthy adults 24 hours after a single intravenous infusion of 0.5, 1, or 2 gm were reported to be 5, 9, and 15 µg/mL, respectively.^27^ A retrospective review of empiric treatment in 1,353 adult patients with melioidosis in northeast Thailand reported that the mortality rate in 528 patients given empiric ceftazidime therapy on admission was 41.7% compared with a mortality rate of 71% in 167 patients who received cefotaxime or ceftriaxone.^28^ This study had several possible confounders; for example, the cefotaxime/ceftriaxone group had a higher rate of positive blood cultures than patients in the ceftazidime group, and this would be predicted to be associated with a higher death rate. However, this finding supports the use of ceftazidime in preference to other third-generation cephalosporins in patients with suspected or confirmed melioidosis.

Ceftazidime has been made available at the AHC and is now the parenteral treatment of choice for children with confirmed melioidosis, followed by a course of oral amoxicillin-clavulanate. Deciding on best practice for empiric treatment is less clear cut, because melioidosis is rare (it accounted for 0.3% of all admissions during the study period), and *B. pseudomallei* ranks low on the list of bacterial pathogens isolated in our laboratory. Ceftriaxone would be the preferred choice over ceftazidime for several of the more common pathogens. Our current policy is to use ceftriaxone as the first-line empiric drug until culture results become available but to use ceftazidime or amoxicillin-clavulanate as the first-line choice in children admitted with acute suppurative parotitis, in cases with multiple abscesses in the liver and/or spleen, and in cases suspected melioidosis based on other grounds. All deaths attributable to melioidosis occurred before culture results became available, and more rapid diagnostic tests are required to improve time to effective therapy. We are reviewing the turnaround time of culture results and the possibility of introducing a direct immunofluorescence test that can be used on pus and other material from suspected cases. This test can provide a presumptive same-day diagnosis and has a reported sensitivity and specificity of 66% and 99.5%, respectively.^29^

The clinical features of the cases described here are very similar to those described elsewhere in Asia. Childhood melioidosis is predominantly a disease of apparently immunocompetent persons, which contrasts with disease in adulthood when at least 80% of those affected have at least one risk factor that affects host immunity. Our finding that two-thirds of disease was localized is consistent with the published literature from Asia,^30^–^34^ and the proportion of localized infections in the region of the head and neck is also consistent.^31^,^33^ A previous report describing acute suppurative parotitis in children caused by *B. pseudomallei* in northeast Thailand suggested that this syndrome may prove to be a sensitive indicator of the presence of melioidosis within a given geographic area.^9^ This has proven to be the case for childhood infection in Asia, although this form of the disease is rare in Thai adults (occurring in 1% of adults with melioidosis in northeast Thailand) and is not observed in Australia.^35^ We speculate that parotitis and infection of the regional lymph nodes of the neck may relate to the presence of contaminated water in the mouth related to bathing, swimming, and drinking of untreated water, whereas localized infection of the lower limbs may relate to inoculation events. Overall death rates in previous pediatric case series have varied from 10–38%, but common to all studies were the findings that no deaths occurred in children with localized disease and that bacteremia was associated with a high mortality rate.^30^–^35^

Melioidosis is not considered in the differential diagnosis in the majority of health-care settings in Cambodia, and the level of local knowledge about this condition is low. Commonly used empiric antimicrobial therapies will rarely include first-line therapy for melioidosis. The identification of melioidosis at the AHC has led to an increased awareness and understanding of this infection in our practice, and this will be followed by improvements in patient care.

## Figures and Tables

**Table 1:** Summary data for 39 children with melioidosis

Variable	Number	
Male gender	15	38%
Age (years), median (range, IQR*)	7.8	1.6–16.2; 4.1–12.4
Underlying disease present	4	10%
Source of *B. pseudomallei* isolate		
Blood†	9	23%
Pus	29	74%
Respiratory secretions	1	3%
Severity of infection‡		
Localized	27	69%
Disseminated	12	31%
Type/site of infection		
Acute suppurative parotitis	15	38%
Superficial soft-tissue abscess	7	18%
Blood culture positive and no focus identified	6	15%
Lymph-node abscess	4	10%
Pneumonia	3	8%
Meningitis	1	3%
Other§	3	8%
Admission WBC (×10^9^ cells); median (range, IQR)	16.7	1.6–33.3; 9.6–20.7
Died during admission	8	21%
Death attributable to melioidosis	7	18%
Time to death (days); median (range)	2	day of admission to day 5

* IQR, interquartile range.

† One child also had *B. pseudomallei* isolated from urine

‡ Localized infection was defined as a single, discrete culture-positive focus of infection in the absence a positive blood culture or clinical and/or microbiological evidence of dissemination to a second site. Disseminated infection was defined as the presence of infection in two or more discrete body sites and/or the presence of *B. pseudomallei* in blood.

§ Psoas muscle abscess (1 case), mastoiditis (1 case), and pharyngeal abscess (1 case).
